# Nanofiber scaffolds based on extracellular matrix for articular cartilage engineering: A perspective

**DOI:** 10.7150/ntno.78611

**Published:** 2023-01-01

**Authors:** Elham Ahmadian, Aziz Eftekhari, Dawid Janas, Parviz Vahedi

**Affiliations:** 1 Kidney Research Center, Tabriz University of Medical Sciences, Tabriz, Iran; 2 Research Center for Pharmaceutical Nanotechnology, Biomedicine Institute, Tabriz University of Medical Sciences, Tabriz, Iran; 3 Department of Pharmacology and Toxicology, Faculty of Pharmacy, Tabriz University of Medical Sciences, Tabriz, Iran; 4 Department of Organic Chemistry, Bioorganic Chemistry and Biotechnology, Silesian University of Technology, B. Krzywoustego 4, 44-100, Gliwice, Poland; 5 Department of Anatomical Sciences, Maragheh University of Medical Sciences, Maragheh 78151-55158, Iran

**Keywords:** Articular cartilage, Scaffolds, Articular cartilage defect, Collagen type II

## Abstract

Articular cartilage has a low self-repair capacity due to the lack of vessels and nerves. In recent times, nanofiber scaffolds have been widely used for this purpose. The optimum nanofiber scaffold should stimulate new tissue's growth and mimic the articular cartilage nature. Furthermore, the characteristics of the scaffold should match those of the cellular matrix components of the native tissue to best merge with the target tissue. Therefore, selective modification of prefabricated scaffolds based on the structure of the repaired tissues is commonly conducted to promote restoring the tissue. A thorough analysis is required to find out the architectural features of scaffolds that are essential to make the treatment successful. The current review aims to target this challenge. The article highlights different optimization approaches of nanofibrous scaffolds for improved cartilage tissue engineering. In this context, the influence of the architecture of nanoscaffolds on performance is discussed in detail. Finally, based on the gathered information, a future outlook is provided to catalyze development in this promising field.

## Introduction

There are three forms of cartilage in the body; hyaline or articular cartilage, fibroelastic cartilage (meniscus), and elastic cartilage 1. Articular cartilage (AC) covers the end of the long bones at the joint place. The main function of AC is to create friction-free movement, reduce load, increase traction, and resist pressure distribution at the joint surface 2. At the same time, it must have high tensile strength, resist lateral tension, and ensure tissue integrity. Also known as hyaline cartilage, it is a unique and durable connective tissue that provides nearly frictionless articulation for mechanical load transmission between joints. Thus, it plays a crucial role in the physiological mobility of joints 3,4.

**Figure 1:**
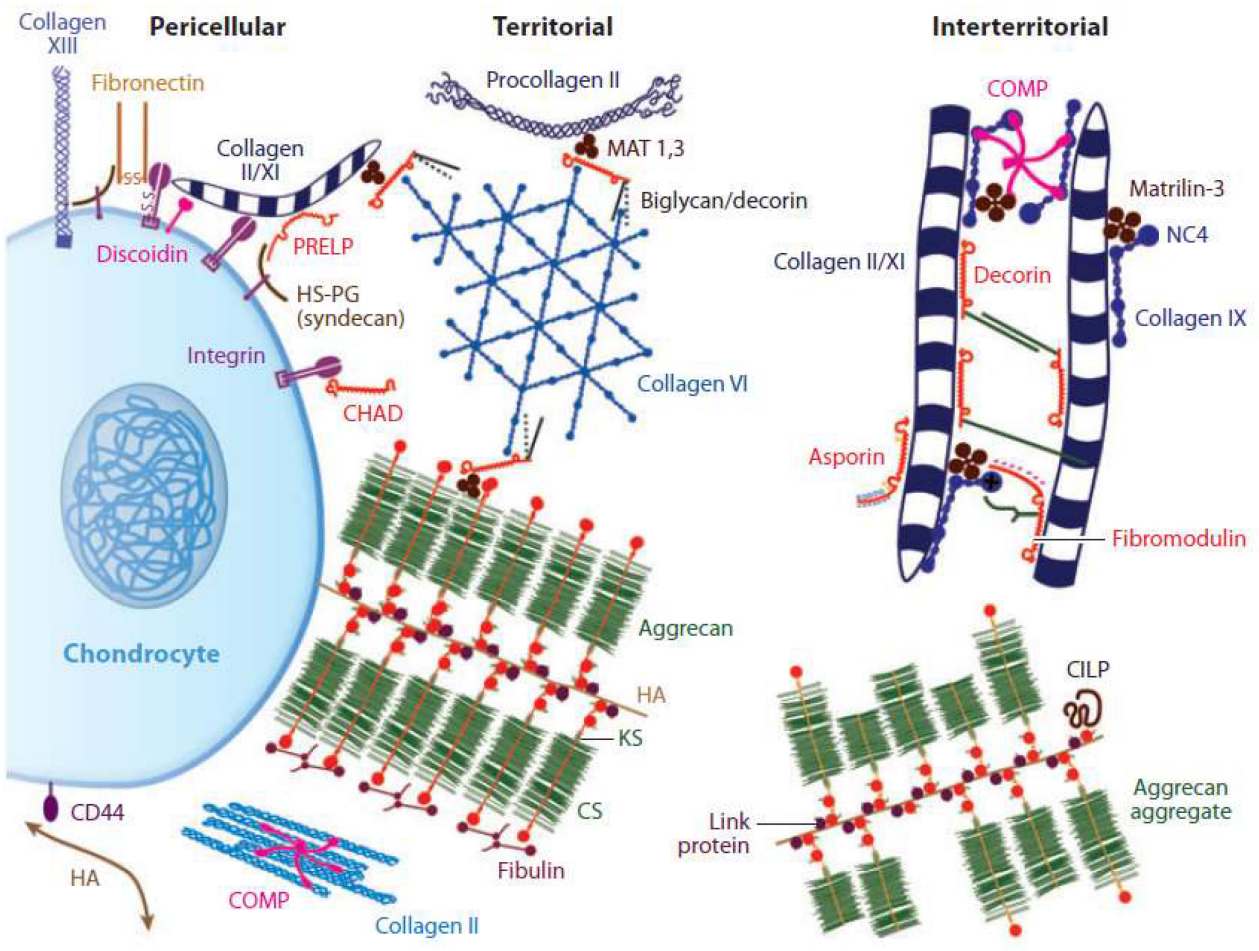
Extracellular matrix of articular cartilage. Two major load-bearing macromolecules are present in articular cartilage: Collagens (mainly type II) and proteoglycans (notably, aggrecan). Smaller classes of molecules, such as non-collagenous proteins and smaller proteoglycans, are present in lower amounts. The interaction between the highly negatively charged cartilage proteoglycans and type II collagen provides the compressive and tensile strength of the tissue. Reproduced with permission 13. Copyrighted by the Authors (2019).

Cartilage architecture predominantly comprises collagen type II, VI, IX, X, chondrocytes, and glycosaminoglycans. Collagen type II mainly forms 90-95% of the fiber network of the extracellular matrix of cartilage and provides its cartilaginous framework and tensile strength 5-8. It exhibits the stress-shielding of the solid matrix components due to its high water content, incompressible under these conditions, and the structural organization of the proteoglycan and collagen molecules 9,10. Furthermore, carbohydrate groups in collagen type II allow more interaction with water than other collagens. This sort of collagen, together with classes IX and XI, forms a fibrous network that leads to the elastic strength of the cartilage 11. Besides these materials, proteoglycans, hyaluronic acid, and heparan sulfate are some of the glycosaminoglycans found in the AC 12. The most prominent proteoglycan is aggrecan, which generates tensile strength, porosity, permeability, and reinforcement together with collagen fibrils in AC (Figure 1) 13.

**Figure 2:**
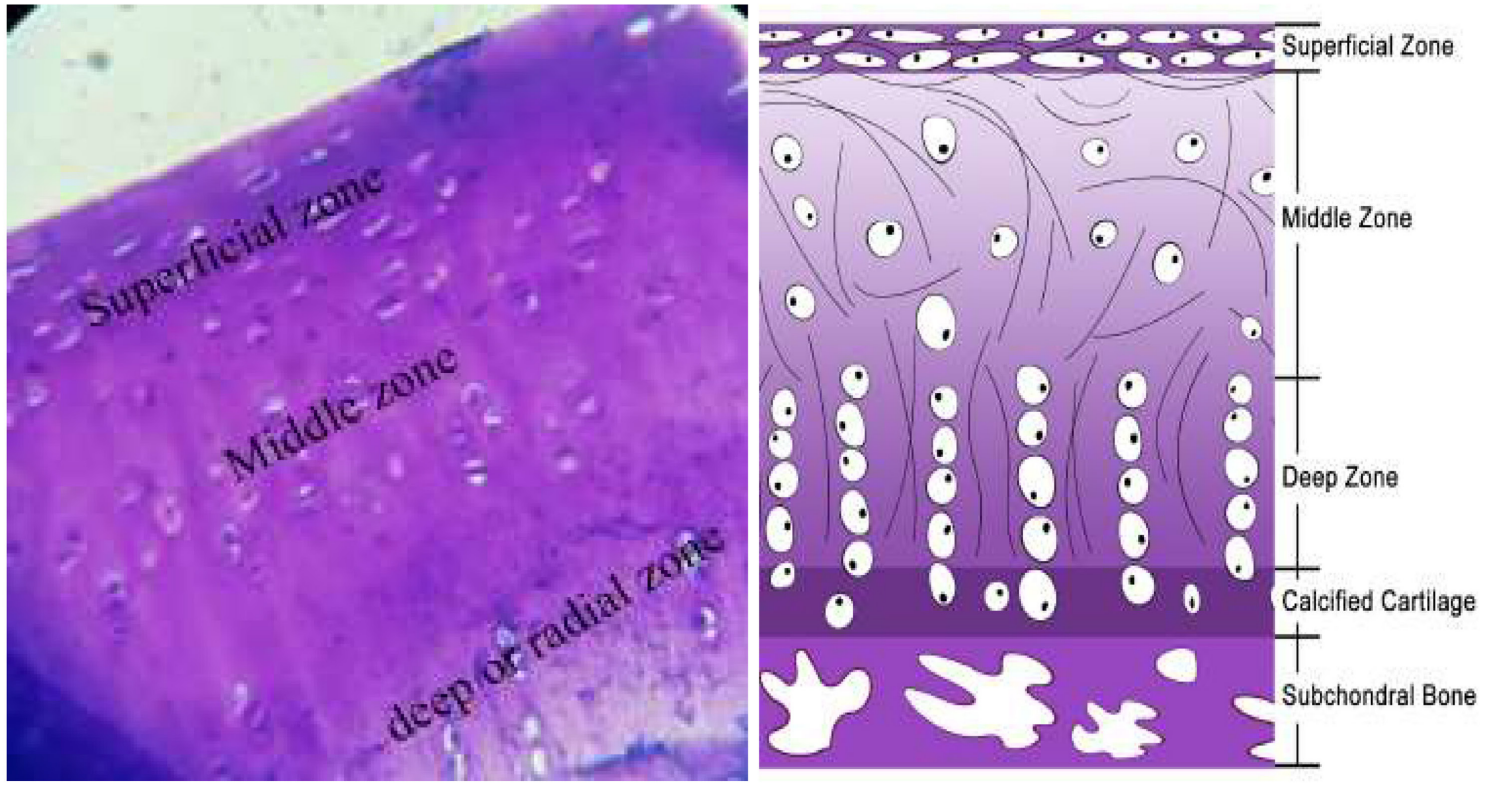
Structure of the articular cartilage layer. Reproduced with permission 20. Copyrighted by the Authors (2021).

AC is divided into three areas: superficial, middle, and deep, each of which has its distinct properties; the number of cells, shape, size, and direction of collagen fibers and proteoglycans (Figure 2) 14-16. The superficial layer of AC is the thinnest, and the elastic properties of cartilage belong to this layer. Chondrocytes and collagen fibers (mainly type II and type IX collagen fibers with an ultra-small diameter (20 nm)) in this layer are aligned parallel to the surface to protect the deeper layers against tensile, compressive, and shear forces 17-19. Given the hierarchical nature of the AC, its repair is non-trivial. This perspective article aims to summarize recent progress in solving this issue using nanoscaffolds and provides directions how to facilitate further development in this field.

## Articular Cartilage damages

AC has a low post-traumatic injury or wear self-repair capacity due to a lack of vessels and nerves. Furthermore, its cell proliferation capacity is not satisfactory 21. Therefore, AC-related defects are especially problematic. In general, people with joint injuries and meniscal or ligament tears are prone to cartilage joint damage 22,23. Consequently, damaged cartilage can lead to arthritis in the joint. Also, AC can be damaged by injury or physiological wear and tear during aging. Initially, in osteoarthritis, chondrocytes release many anabolic factors to repair the lesions. However, the factors alter the phenotype of chondrocytes by forming non-functional cartilage (fibrocartilage), which is thinner than regular AC and degenerates under mechanical pressure 24.

## Treatment procedures for articular cartilage defects

Treatment of AC injuries is one of the most challenging issues of musculoskeletal medicine due to the poor intrinsic ability of this tissue to repair itself. Nevertheless, restoring AC is essential as it can relieve pain and improve function. Most importantly, it can delay or prevent the onset of arthritis. The most common procedures for cartilage restoration are as follows.

### Microfracture

Microfracture is commonly used for cartilage restoration by marrow stimulation. This method increases the migration of mesenchymal stem cells (MSCs), the influx of growth factors, and platelets from bone marrow to the defect site. However, this method results in the formation of fibrocartilage rather than normal hyaline AC 25, which is unwanted.

### Drilling

This therapeutic approach creates holes in the subchondral bone and supplies blood flow into defects to induce the repair of cartilage. The holes are made with a surgical drill or wire. Although the method is similar to microfracture, in this technique, the heat of the drill damages the AC tissues. It again results in the formation of fibrocartilage 26,27, so it is also not recommended.

### Abrasion Arthroplasty

Abrasion Arthroplasty removes some areas of the AC to create new capacities for joint surfaces. Hence, it can eliminate the injured cartilage tissue 28. But unfortunately, this is only a palliative approach as it does not promote AC regeneration. Consequently, it is employed as a last resort treatment.

### Autologous chondrocyte implantation (ACI)

Autologous Chondrocyte Implantation (ACI) is used to repair AC defects in a surgical approach. ACI is divided into a two-step procedure. In the first surgical procedure, healthy cartilage tissue is taken from a non-weight-bearing area of the joint during an arthroscopic procedure. The cartilage tissue is then sent to the laboratory, and chondrocytes are isolated, which are subsequently cultured and proliferated. The second surgical procedure (arthrotomy) is an open procedure in which the newly grown cells are injected into the articular cartilage defects 29. ACI is the most useful technique for treating isolated cartilage defects in younger patients with larger defects in AC who would like to return to activities of daily living. Unfortunately, despite its merits, this approach does not restore the possibility of competing in high-level sports 30-32.

### Osteochondral Autograft Transplantation (OCA)

**Figure 3:**
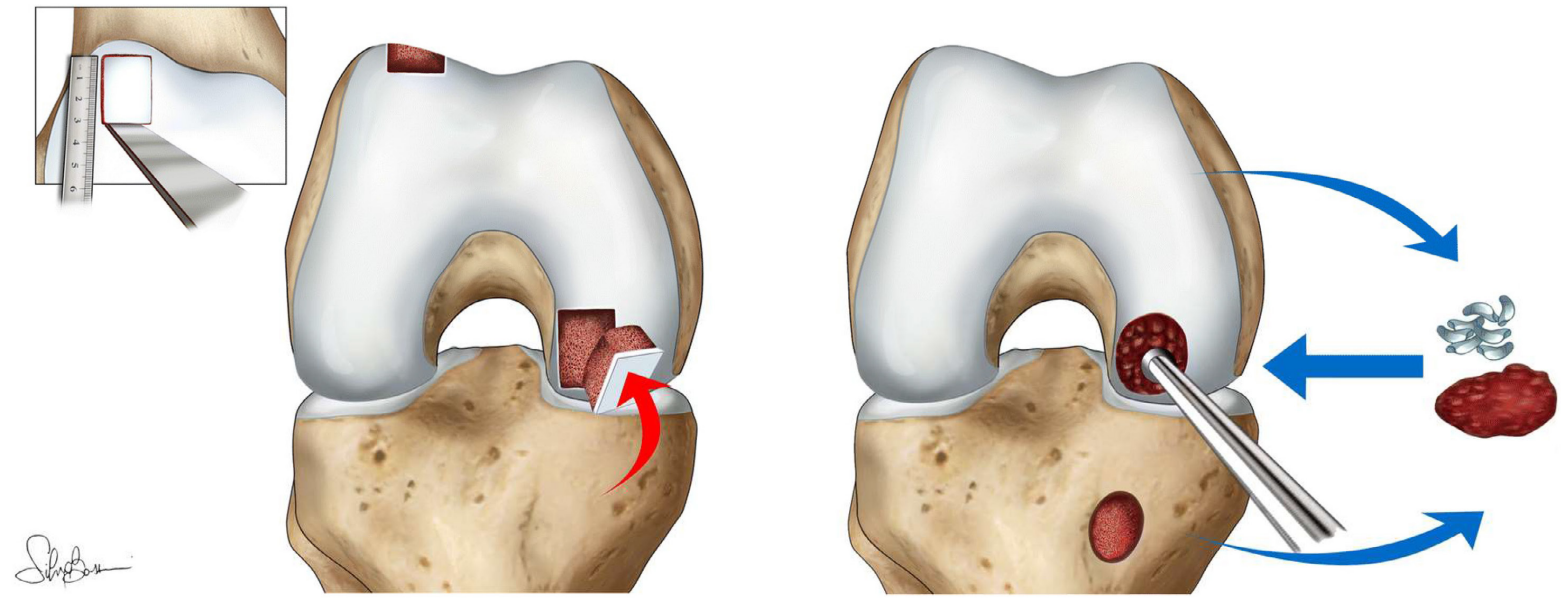
Schematic representation of osteochondral autograft transplantation. Reproduced with permission 35. Copyrighted by the Authors (2021).

OCA has demonstrated consistent clinical results and can be used to treat a variety of articular defects of the knee using size-matched cadaveric donor plugs that permit immediate structural restoration of the joint articular surface 33,34 (Figure 3).

All described treatment strategies have been applied to regenerate cartilage lesions to restitute articular function and relieve the associated pain. Although they decrease patient discomfort and enhance joint mobility, the repaired tissue is often fibrocartilage with less clinical action 36.

## Engineering articular cartilage tissue

Tissue engineering is used to reconstruct and regenerate damaged tissues. It is a medical technology that has attracted considerable attention in recent times. The main purpose of this approach is not only to repair tissue but primarily to improve organ function. Tissue engineering also has diagnostic applications made *in vitro* and is used to test the biocompatibility of the materials 37. Three components are required to achieve an ideal tissue engineering procedure: appropriate scaffold, cell, and induction factors. In recent decades, success in tissue engineering has mainly influenced the therapy of defects and tissue regeneration. A stable increase in life expectancy and a related improvement in life quality are linked with this development, including the understandable request of the population to accept no damage in increasing age during the whole life. It is evident that modern medicine seeks solutions to avoid the loss of cell or tissue functions 3,38, which is more beneficial than treating advanced state diseases.

## Nanofiber scaffolds

Since the current surgical procedures for regenerating a cartilage defect have considerable drawbacks, 3D scaffolds might offer promising results to facilitate the restoration of target tissues 39. Also, the advances in nanotechnology have offered groundbreaking progress in the field of tissue engineering by providing a microenvironment for the induction of cell expansion and differentiation into the desired lineage 40. The development in this area is particularly dynamic as nanomaterials exhibit good physicochemical and biomimetic properties that can stimulate the growth of chondrocytes and the regeneration of AC 41. Nanofibers have been considered one of the most extensively studied nanomaterials for cartilage regeneration and are constructed via different techniques, including electrospinning, self-assembly, phase separation, and drawing 42-44. The polymer nanofibers used for the regeneration of AC are both synthetic and natural scaffolds. Natural nanofiber scaffolds include alginate 45, gelatin 46, agarose 47, hyaluronic acid 48, fibrin, and collagen 49. Synthetic scaffolds are very diverse and mostly contain polycaprolactone 50, polyethylene glycol, polyurethane 51, poly(p-dioxanone) 52, poly(lactic acid) 53. It is of utmost importance to ensure that the selected scaffold exhibits tissue compatibility and biodegradability.

On the one hand, synthetic polymers are reproducible, and their properties can be easily controlled 54. Many polymer nanofibers well maintain cartilage stem cell differentiation and proliferation *in vitro* 55,56. Nevertheless, Earth-abundant natural polymers have more applications in clinical studies, and collagen is the most prevalently used type 48. Collagen polymers provide an essential network 57 for cartilage formation, both with and without cells 58. In general, natural scaffolds are used as gels in which the polymer network retains a large amount of water. This environment supports the proliferation, differentiation, and adhesion of cells. However, unfortunately, these polymers have poor mechanical properties and weak resistance to pressure and tension 59.

Regardless of the origin, the nanofiber scaffolds should stimulate the growth of new tissue in all aspects and mimic the characteristics of AC and the area below the articular cartilage. In addition, the scaffold should exhibit the potential to merge with the target and withstand physical activity tissue 60.

## The effect of architectural features of scaffolds on cartilage repair

**Figure 4:**
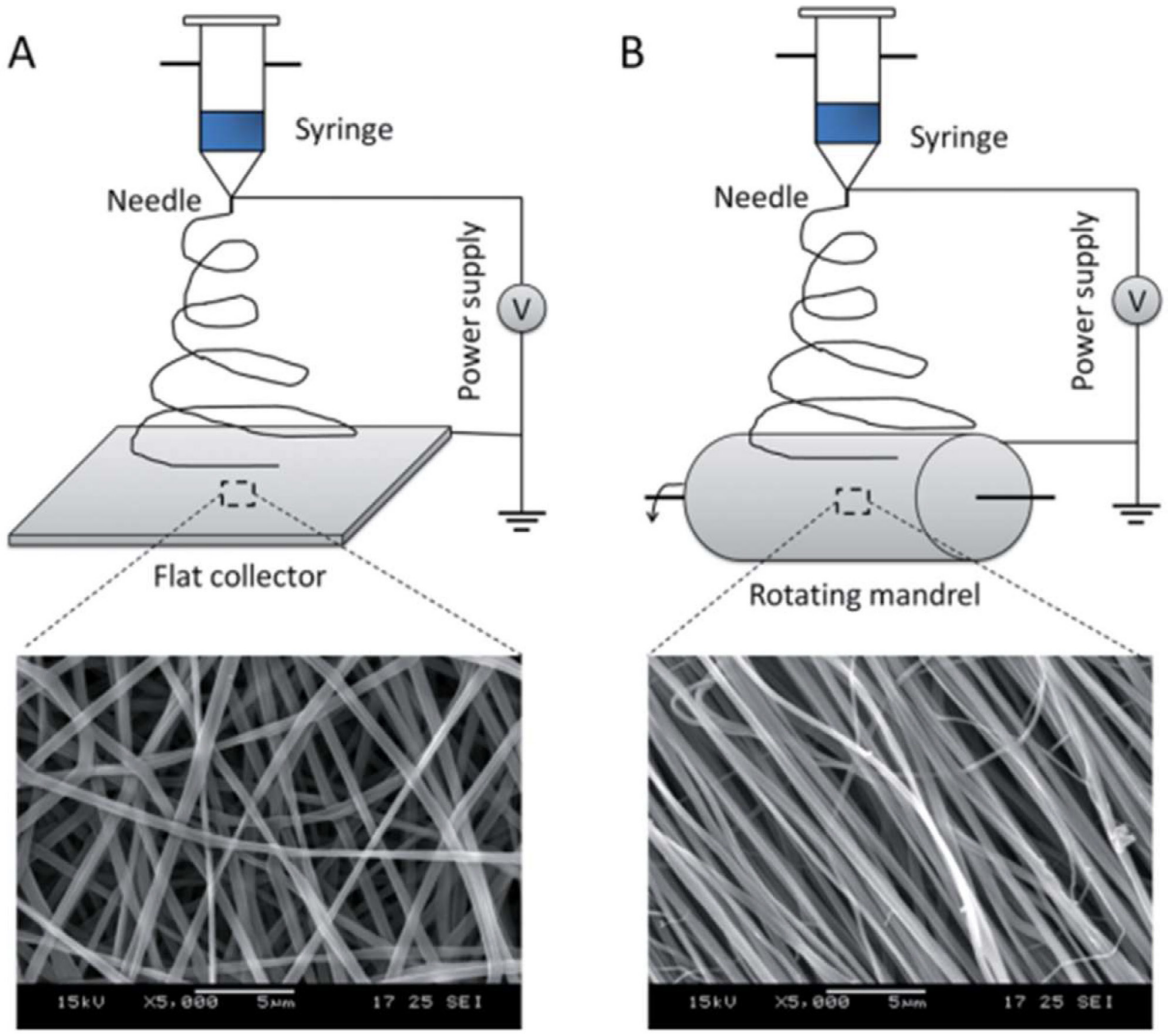
Electrospinning of fibrous (A) isotropic and (B) anisotropic nanoscaffolds. Reproduced with permission from 61. Copyrighted by Wiley (2012).

Scaffolds have been applied to improve tissue regeneration by recapitulating a difference in the physical structure of the tissue. Different strategies have been used, including fibrous self-synthesis, phase segregation, and electrospinning of nanofibers to produce the fibrous collagen network of the extracellular matrix via the aid of nanotechnology. Today, in reconstructive medicine, electrospinning scaffolds are used to repair and regenerate the tissues, such as heart muscle, cartilage, bone, nerve, and others. Interestingly, optimization of the nanoscaffold synthesis conditions may even lead to fiber alignment to obtain anisotropic materials for cell growth (Figure 4) 61.

Some research has shown the potential of electrospinning of fiber scaffolds for cartilage tissue restoration 62. Different 2D electrospun nanofibrous matrices containing a single polymer have been implemented in the chondrogenic differentiation of BM-derived SCs 63. It has been shown that soluble factors minimally affect a cellular orientation, and mainly physical cues are connected with this issue. However, chondrogenic factors influence cell shape, which should be taken into account 64. The expression of collagen type II and S GAG content has shown a substantial increment in cells cultured on the nanofibrous scaffold in the presence of chondrogenic media.

In another study, the culture of MSCs on electrospun PCL nanofiber stimulated the fibroblast-like morphology. In contrast, the dynamic condition led to a round-shaped morphology with elevated collagen type I, II, and sGAG 65. Dahl et al. reported the effects of PLGA/PCL electrospun nanofibers on chondrogenic differentiation of human Umbilical Cord MSCs 66, resulting in high levels of proteoglycans and sGAG.

Other hybrid nanofiber scaffolds have also been applied in this context. For instance, PLGA/collagen scaffolds have shown optimal chondrogenesis potential 52. Li et al. have developed a PLLA/silk fibroin (PLLA/SF) composite that provided a suitable platform for the adhesion and growth of chondrocytes for cartilage tissue engineering purposes 67. Aligned nanofibrous scaffolds have been shown to mimic the naturally-occurring extracellular matrix and result in fibrochondrogenesis 68. Moreover, Shafiee et al. have shown that aligned PLLA/PCL nanofibrous scaffolds and compared them with randomly oriented scaffolds. The results showed that aligned scaffolds led to a bipolar extension along the fiber and a significantly higher expression of the chondrogenesis markers 69.

Although nano-sized structures simulate well the ECM components, they can also promote cell spreading and restrict infiltration 70,71. Thus, the construction of micro-nanofibers can overcome these limitations and assist in yielding larger pore sizes, improved cellular differentiation, and formation of ECM 72. Leverson et al. have fabricated electrospun PCL microfibers (Pμ), PCL microfibers with PCL nanofibers (PμPn), and PCL microfibers and fibrin nanofibers (PμFn) scaffolds. Accordingly, similar porosity was observed in both PμFn and PμPn scaffolds. However, larger pore sizes were obtained in Pμ scaffolds. Also, higher density was observed in PμPn scaffolds. More elongated spindle-like cells of human umbilical MSCs appeared on Pμ and PμFn scaffolds, whereas flattened, broad polygonal morphology was observed for PμPn scaffolds 73.

The synthesis of 3D nanofibrous scaffolds has also highly enhanced the chondrogenesis of different SCs 74. Li et al. have shown that a 3D nanofibrous scaffold produced a higher level of cartilaginous ECM from chondrocyte-like cells 75. Moreover, it has been revealed that biomolecules influence the chondrogenic differentiation of SCs seeded on nanofibrous structures. Schagemann et al. have reported that augmentation of the nanofibrous scaffold with or without TGF-β1 and/or hyaluronan could positively affect the chondrogenic differentiation of MSCs. Also, they showed that microfibrous scaffolds release higher amounts of TGF-β1. However, these scaffolds show a lower level of chondrogenic markers compared to nanofibrous scaffolds 76.

Research has also shown that scaffolds with parallel fibers and random Poly(l-lactide) (PLLA)/Polycaprolactone (PCL) 69 and Polycaprolactone (PCL)/Poly(lactic-co-glycolic acid) (PLGA) can support progenitor cells and human bone marrow-derived stem cells, adhesion, proliferation, and regeneration of cartilage 77. However, the proliferation of stem cells was higher on the random scaffolds than on the aligned scaffolds. In contrast, the differentiation of stem cells was higher on the scaffolds with parallel fibers. Thus, such a scaffold with paralleled fibers structure is suitable for the superficial area of AC wherein its fibers are arranged parallel to the surface.

**Figure 5:**
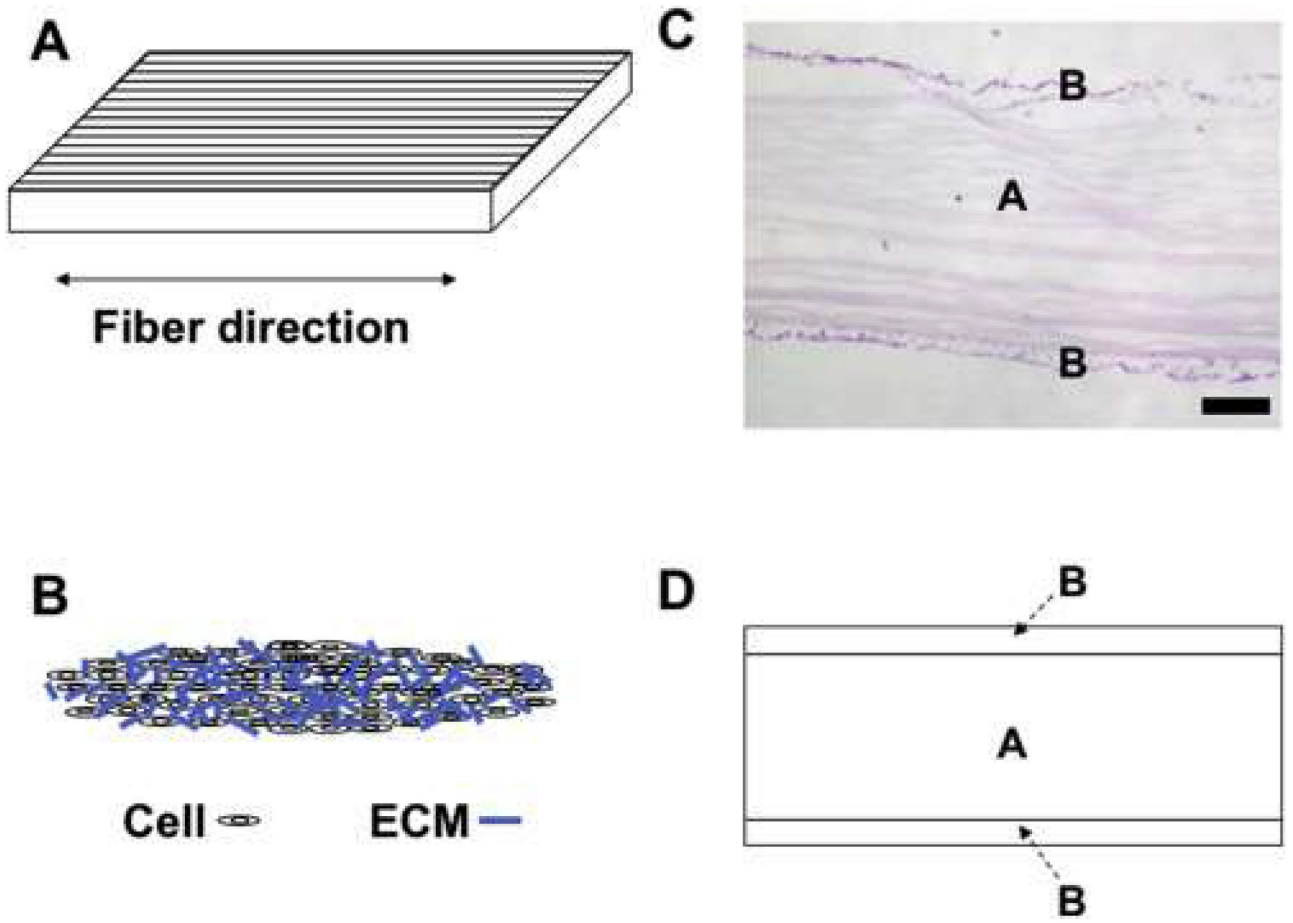
Schematic illustration of a mixed material of a nanofiber scaffold along with scaffold-free tissue-engineered construct (TEC) consisting of cells and extracellular matrix (ECM). A: The parallel structure of the scaffold. B: TEC structure. C: H&E staining of the combined material consisting of an electrospun nanofibrous scaffold (A) and a TEC (B). D: Schematic depiction of combined material comprising an electrospun nanofibrous scaffold (A) and a TEC (B). Reproduced with permission 84. Copyrighted by the Authors.

Although nano- and microfibers electrospun scaffolds maintain and support the growth and proliferation of bone marrow-derived stem cells, nano-fibrous PCL scaffolds play a greater cartilaginous activity. That was shown by produced sGAG and collagen type II expression, and this scaffold may be suitable for the repair of the superficial area of the scaffold. Other studies have reported similar results with nanofiber scaffolds, as opposed to microfiber scaffolds or smooth (film) Poly(L-lactide) (PLLA) 78 or poly (L-co-D,L-lactide) (PLDLA) 79 scaffolds. The nano-fibrous structures preserve the morphology of chondrocytes and increase the expression of cartilage markers and the formation of the extracellular matrix. Nevertheless, it should be noted that not only the fiber size and porosity but also the pore size acts as a significant factor in carcinogenesis 80. Electrospun nanofiber scaffolds with low porosity/high density often cause negligible cell permeability and limit nutrient infiltration to the deep zone of the cartilage tissue 81. Thus, various modifications in the fiber size and density have been applied to strengthen and increase the permeability and transport potential of biomaterials through scaffold fibers 82,83. Paralleled PCL strands combined with synovial-derived stem cells have shown a significant repair of meniscal hoop structure injuries in rabbits 84 (Figure 5).

In swine, as a large animal, the implantation of PCL/BMSCs in full-thickness cartilage defect of AC led to a complete repair and formation of hyaline cartilage-like tissue 6 months after seeding 85. Seeding collagen/PLCL scaffolds with chondrocytes in layer-by-layer sandwich constructs of collagen/PLCL in mice harvested 83% of native cartilage after 12 weeks of transplantation 86. PVA/chondroitin sulfate electrospun fibers seeded in a rat articular cartilage defect showed increased chondrogenesis compared to the control group of the empty defect 87. Resveratrol-PLA-gelatin scaffolds, compared with PLA-gelatin scaffolds, were found to treat faster the rat articular cartilage defect 12 weeks post-transplantation 88. Repairing the cartilage defects in the rabbit model by aligned PLLA-polydopamine chondroitin sulfate fibers was facilitated, and cartilage defects were regenerated by hyaline cartilage-like tissue 89. In light of the foregoing, the application of nanoscaffolds for cartilage restoration is an auspicious approach with a high application potential, which can gain from recent breakthroughs in biomedical engineering 90-93.

## Conclusion and Future Outlooks

This review discussed the application of nanofiber scaffolds that can result in the complete synthesis of native hyaline cartilage tissue in optimum situations. Tissue replacement through non-biological and regenerative systems *in vivo* and the formation and design of nanofiber scaffolds are currently hot topics in the tissue engineering field because of its merits. Still, the examined literature reveals increasing demand for qualified tissue regeneration strategies to increase the number of successful transplantations. Thus, it is vital to develop strategies for creating cells, tissues, and organs with good compatibility to reduce the future rejection rate of clinical programs 94. In light of the presented research, tissue engineering appears to be a promising approach to reach these goals. Furthermore, judging by the maturity of the results, the production of appropriate scaffolds for tissue engineering may soon materialize at a sufficiently large scale 95. Nevertheless, the multidisciplinary nature of this challenge hampers progress. Thus, it is necessary to promote interactions between engineers and physicians to provide possibilities for their collaboration for the sake of tissue engineering.

**Figure 6:**
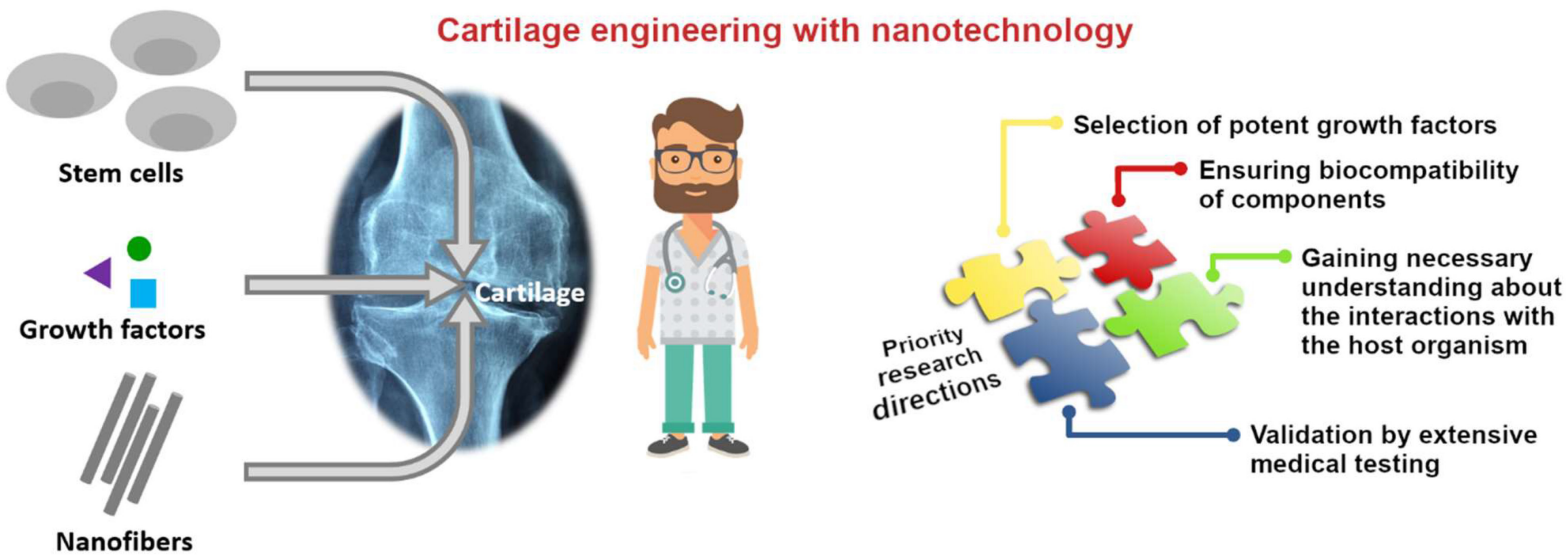
Most important factors, which should be the matter of further research to increase the technology readiness level of nanofiber-bases scaffolds for cartilage engineering.

Importantly, there is much room to optimize the performance of nanoscaffolds. For instance, future scaffold constructs will only be successful if improved compatibility regarding the ECM is realized 96. In addition, a broader spectrum of nanofibers, stem cell sources, and differentiation-inducing growth factors should be evaluated to reach the most suitable combination of materials for cartilage repair (Figure 6).

In this context, the fabrication and implantation of appropriate bioreactors that eventually aid in more ECM formation and the achievement of artificial constructs mimicking native cartilage tissues should be considered. Finally, although several investigations produced promising results regarding the efficacy of nanofibers for repairing cartilage defects *in vivo*, more effort is still necessary for shifting the information from *in vitro* to *in vivo* phase. Only then this promising concept will serve society and improve the quality of our life.
